# Comparative Analysis of β-Carotene Production by *Mucor circinelloides* Strains CBS 277.49 and WJ11 under Light and Dark Conditions

**DOI:** 10.3390/metabo10010038

**Published:** 2020-01-16

**Authors:** Tahira Naz, Shaista Nosheen, Shaoqi Li, Yusuf Nazir, Kiren Mustafa, Qing Liu, Victoriano Garre, Yuanda Song

**Affiliations:** 1 Colin Ratledge Center of microbial lipids, Shandong University of Technology, Zibo 255000, China; nazkhan658@gmail.com (T.N.); shaista_nosheen@yahoo.com (S.N.); lsq1639471373@outlook.com (S.L.); yusufnazir91@yahoo.com (Y.N.); mustafakiran92@gmail.com (K.M.); qingliu0906@sdut.edu.cn (Q.L.); 2 Departamento de Genética y Microbiología (Unidad asociada al IQFR-CSIC) and Facultad de Biología, Universidad de Murcia, 30100 Murcia, Spain; vgarre@um.es

**Keywords:** β-carotene, *Mucor circinelloides* strains, mevalonate pathway, transcriptional levels

## Abstract

Carotenoids are natural potent antioxidants and free radical scavengers which are able to modulate the pathogenesis of some cancers and heart diseases in human, indicating their importance in being provided through the diet. *Mucor circinelloides* accumulates β-carotene as the main carotenoid compound and has been used as a model organism in carotenogenic studies. In the present study, the potential of two *M. circinelloides* strains to accumulate β-carotene was investigated under light and dark conditions. The results, which were quantitated by HPLC, showed that CBS 277.49 accumulated higher pigment in comparison to WJ11 under both conditions. Continuous illumination triggered the pigment accumulation up to 2.7-fold in strain CBS 277.49 and 2.2-fold in strain WJ11 in comparison to dark. The mRNA analysis of the four key genes involved in isoprenoid pathway by RT-qPCR showed higher transcriptional levels in CBS 277.49 as compared to WJ11, indicating that the pigment production metabolic machinery is more active in CBS 277.49 strain. A new scope for further research was established by this work for improved β-carotene production in the high producing strain CBS 277.49.

## 1. Introduction

Carotenoids are one of the most important groups of natural antioxidants and have been extensively utilized in food, pharmaceutical, and cosmetic industries. Their antioxidant property can be applied in boosting the immune system [1] and prevention of different types of cancer in humans and animals by scavenging the reactive oxygen species (ROS) molecules. Up till now, over 600 carotenoids have been identified in nature, but only a few are of industrial interest (β-carotene, astaxanthin, lutein, or lycopene). Many industries produce carotenes chemically, but due to safety concerns regarding artificial food coloring, there is increased interest in carotenoids produced by biological sources [2]. β-carotene is a lipophilic carotene, considered as pro-vitamin A, and is of great commercial value due to its usage in pharmaceutical products, cosmetics, food, and textiles, with diverse biological functions [3]. According to one report, the global β-carotene market size in 2017 was over USD 439.0 million, and it is more likely to grow at a “Compound annual growth rate (CAGR)” of more than 3.8% and expected to reach USD 618.94 million by 2026 (https://www.polarismarketresearch.com/industry-analysis/beta-carotene-market). Major benefits of β-carotene include eye health, healthy skin, and prevention from cardiovascular diseases. Its rich pigmentation level makes it a suitable compound for use in the pharmaceutical, food, and beverage industries.

Three zygomycetes, i.e., *Phycomyces blakesleeanus*, *Blakeslea trispora*, and *Mucor circinelloides*, have been reported for their capacity to synthesize carotene, in which β-carotene is the predominant carotenoid species [4,5]. *P. blakesleeanus* and *B. trispora* have been reported as highest producers of carotenoid and used industrially, but there are few drawbacks associated with the industrial use of these two zygomycetes. For instance, shaking cultivation of *B. trispora* has been accompanied with a decline in carotenoid accumulation, so surface cultivation must be employed; and the optimal growth temperature for these two fungus should never be above 25 °C [6]. Furthermore it has been suggested recently that industrial production of carotenoid using *M. circinelloides* could be much simpler than the complex fermentation process used for β-carotene production by *B. trispora* [7]. *M. circinelloides* is more favorable among other zygomycetes for genetic studies due to the availability of molecular tools, as well as its genomic sequence (http://www.genome.jgi.doe.gove/Mucci2.home.html). *M. circinelloides* is also an attractive target for further biotechnological developments, owing to the availability of an efficient transformation system and its capacity to express exogenous genes, allowing improved carotene-content production [2,5].

*Mucor* genus is an early diverging fungal lineage, which belongs to phylum mucormycota and mucorales order among filamentous fungi [8]. *Mucor* genus is widespread and common in nature [9,10] due to its rapid growth and high sporulation, which makes it ubiquitous, except its inability to grow on substrates with low water activities (aw). Due to increased biotechnological interests, several *Mucor* species have been considered as a potential candidate for secondary metabolite production and considered as good source of lipids, ethanol, food colorants, cellulases, phytases, and proteases [11]. Production of these metabolites is not a normal physiological phenomenon and generally requires environmental stress (light) or nutritional stress (e.g., N or P limitation) for the accumulation of these metabolites (carotenoid and lipid) in the mycelium [12,13]. Zhang et al., reported that carotenoid and lipid production requires excess carbon and limited nitrogen, which might result in inhibition of cell growth and propagation [14]. In this process, accumulation of lipid functions as an energy sink, and carotene works as an electron sink when nutrient limitation causes an energy imbalance in microorganisms [15].

**Figure 1:**
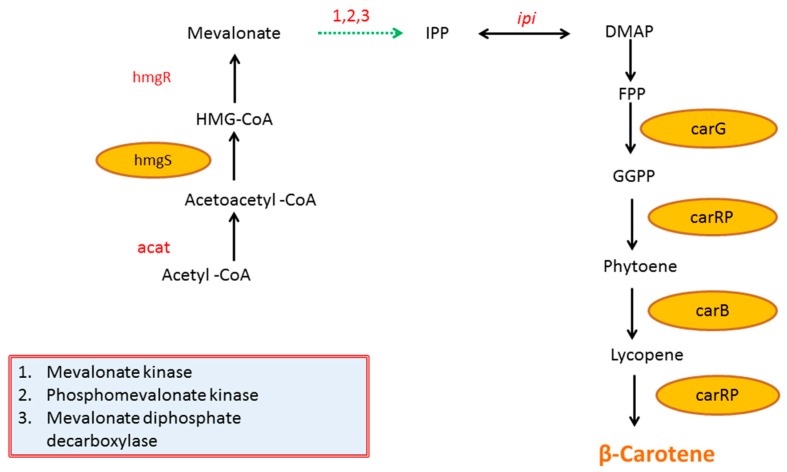
Metabolic pathway of β-carotene biosynthesis in *M. circinelloides*. Dashed lines represent multiple steps. HMG-CoA-3-hydroxy-3-methylglutaryl-CoA; IPP-isopentenyl pyrophosphate; DMAP-dimethylallyl pyrophosphate; FPP-farnesyl pyrophosphate; GGPP-geranylgeranyl pyrophosphate. *M. circinelloides* genes investigated in present study are highlighted in yellow: *acat*-Acetyl-CoA thiolase; *hmgS*-HMG-CoA synthase; *ip*i-IPP isomerase; *carG*-GGPP synthase; *carRP*-phytoene synthase/lycopene cyclase; *carB*-phytoene desaturase [2].

Carotenoids’ production in *M. circinelloides* is regulated by complex genetic mechanisms, and they are derived from 3-hydroxy-3-methylglutaryl-CoA (HMG-CoA), which serves as the first precursor for mevalonate synthesis. This HMG-CoA is produced from acetoacetyl-CoA in a process which is catalyzed by HMG-CoA synthase (*hmgS*). The carotenoid-specific biosynthesis starts with the condensation of two 20-carbon GGPP units [16]. Geranylgeranyl pyrophosphate synthase (*carG*) is also a key enzyme of the carotenoid pathway that carries out the conversion of farnesyl pyrophosphate (FPP) into geranylgeranyl pyrophosphate (GGPP) [17]. This conversion is considered as the first bottleneck in mevalonate pathway, since *carG* was found to be co-regulated with the structural genes (*carB* and *carRP*) of the carotenoid biosynthesis pathway [18]. Genetic analysis of carotene mutants in *P. blakesleeanus* found only two structural genes, *carB* and *carR*, are responsible for the conversion of phytoene to β-carotene [19,20]. In *M. circinelloides*, these two genes are “*carB* (Phytoene dehydrogenase)” [21] and “*carRP* (phytoene synthase/lycopene cyclase)” [22], which carry out the conversion of Phytoene to lycopene and then from lycopene to β-carotene, respectively (Figure 1).

Light plays a very important role in the regulation of developmental and physiological processes in several organisms, including filamentous fungi such as *P. blakesleeanus, N*. *crassa* and *M. circinelloides*. The light response in *M. circinelloides* and *P. blakesleeanus* for carotenoid biosynthesis is well-known [18,23]. Transcriptional analysis of structural genes of β-carotene biosynthesis (*carB* and *carRP*) showed increased mRNA levels in response to light, in accordance with the improved carotene content [7]. In *N. crassa*, the most prominent carotenogenic synthesis by light was observed [24,25]. Upon exposure to blue light, the genes involved in carotene production were upregulated, leading to fast accumulation of orange-color carotene pigment [26] as compared to mycelia that were grown in the dark which had only trace of carotenes. Similarly, it has been demonstrated that carotene production was stimulated under continuous illumination in *Fusarium oxysporum* [27].

In the present study, two strains of *M. circinelloides* CBS 277.49 and WJ11 were selected for comparative study of β-carotenoid production under light and dark conditions. These strains were reported as an excellent source of lycopene, β-carotene, lipids, and other bioactive compounds [7,28]. It was previously demonstrated that lipid accumulation is correlated with an increase in carotenoid accumulation [13] so we speculated that this high lipid producing strain [29] might produce high amount of β-carotene. Many studies have already been done on β-carotene production in *Mucor* but to date, no one used this WJ11 strain for carotenoid study. This is the first study to report the carotenogenesis within this strain (WJ11). Lipid and β-carotene are initially produced from the same pool of acetyl-CoA and then further proceed through FAS and Mevalonate pathway [30]. To determine how does upregulation of the mevalonate pathway under light affected mevalonate flux, the expression of key genes *hmgS*, *carG*, *carB*, and *carRP*, involved in β-carotene biosynthesis were quantified by reverse-transcription quantitative PCR (RT-qPCR). The effect of light in the regulation of expression of these four genes in both strains of *M. circinelloides* was investigated and their collaborative upregulation in response to light was also demonstrated.

## 2. Results and Discussion

### 2.1. The Impact of Light and Dark Conditions on Cell Dry Weight (CDW) of CBS 277.49 and WJ11 Strain

**Figure 2:**
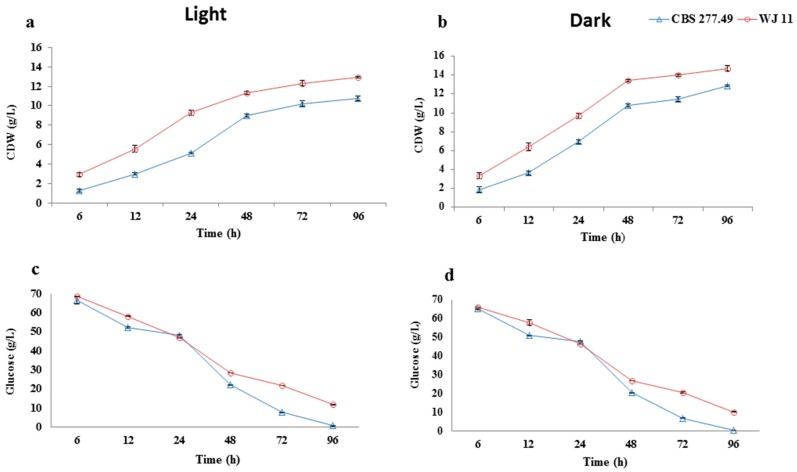
Cell dry weight of *M. circinelloides* CBS 277.49 and WJ11 cultivated in 2 L fermenter with 1.5 L modified K&R medium. (**a**) Light condition; (**b**) dark condition; (**c**) glucose concentration in media (light); and (**d**) glucose concentration in media (dark). Values are mean ± standard deviation of at least three independent experiments. Error bars represent the standard error of the mean.

CDW of *M. circinelloides* CBS 277.49 and WJ11 grown under light and dark conditions were measured (Figure 2a,b), and the results showed that both strains exhibited a similar and typical growth profile. The CDW was increased with cultivation time, till 96 h, which was consistent with the decrease of residual glucose concentration (Figure 2c,d) in both conditions, i.e., light and dark. The glucose consumption was a bit more by both strain in dark condition in comparison to light for both strains. But the growth in light condition was slightly affected as compared to culture grown in dark for both strains, which might be attributed to light stress in our study. This result was contrary to the results reported by Zhang et al., as they found an increased growth rate in *Rhodotorula glutinis* upon continuous illumination [14]. The CDW increased rapidly from 0 to 48 h and then gradually increased in both strains. We found that CDW of WJ11 was higher in comparison to strain CBS 277.49 in both light and dark conditions. This was in accordance to the findings of Tang et al., which indicated high biomass production in WJ11 than CBS 277.49 [29]. After 48 h, the growth of strain CBS 277.49 and WJ11 increased gradually, reaching its maximum stage at 96 h, 10.8 and 12.9 g/L in light, and 12.9 and 14.7 g/L in dark, for CBS 277.49 and WJ11, respectively (Figure 2a,b).

### 2.2. Mycelia Appearance and Analysis of β-Carotene Accumulation by HPLC

**Figure 3:**
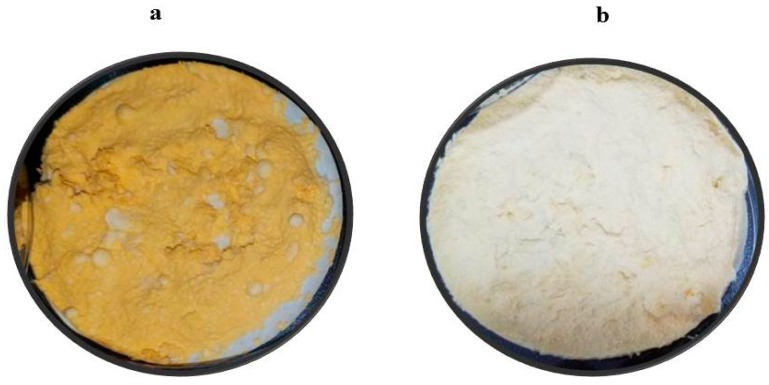
Mycelia appearance of (**a**) CBS 277.49 and (**b**) WJ11 after vacuum filtration and lyophilization.

The mycelium color for both CBS 277.49 and WJ11 strain were light yellow to white when grown in the dark condition, but, upon exposure to continuous illumination, the mycelium color turned to bright orange (CBS 277.49) and deep yellow (WJ11), giving the indication of carotenoid accumulation in both fungal strains (Figure 3). In order to validate the macroscopic observation of the fungal mycelium, analysis of β-carotene production was carried out by HPLC.

HPLC analysis of standard showed that the retention time of β-carotene was 11.32 min, and the peaks of the extracted β-carotene from the two strains of *M. circinelloides* appeared at the same retention time. Quantification of β-carotene by HPLC showed a gradual increasing trend of β-carotene accumulation by strain CBS 277.49 and WJ11, till 48 h, when grown in light and dark conditions. Maximum amount of pigment was produced by both strains at 48 h, after which its production slowed down. 

**Figure 4:**
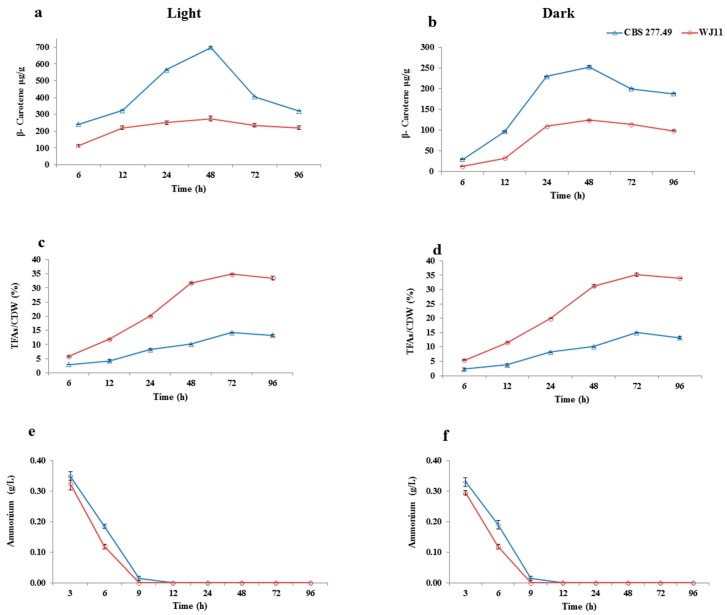
β-carotene accumulation (μg/g), Total fatty acid % and ammonium concentration (g/L) in CBS 277.49 and WJ11, cultivated in 2 L fermenter with 1.5 L modified K&R medium. (**a**) β-carotene production in light, (**b**) β-carotene production in dark condition, (**c**) total fatty acids (TFAs) content (w/w) in light, (**d**) total fatty acids (TFAs) content in dark, (**e**) ammonium concentration in medium under light, and (**f**) ammonium concentration in medium under dark. Values are mean ± standard deviation of at least three independent experiments. Error bars represent the standard error of the mean.

As shown in Figure 4b, the production of β-carotene from 0 to 6 h was not significant in dark condition for CBS 277.49 (29.3 μg/g ± 0.95) and WJ11 (12.5 μg/g ± 0.71). However, upon the depletion of nitrogen content in the media, both strains started to accumulate β-carotene substantially, achieving maximum accumulation at 48 h of cultivation: 252.9 ± 1.55 μg/g (CBS 277.49) and 123.9 ± 0.9 μg/g (WJ11). This observation under the dark condition might be attributed to the depletion of nitrogen content in the media (Figure 4f), resulting in increased accumulation of acetyl-CoA pool in the cytosol of the fungal strains, which is an important precursor for both lipid and β-carotene biosynthesis [13,31], leading to increased accumulation of the β-carotene in CBS 277.49 as compared to WJ11in our study. It was previously reported that nitrogen limitation significantly enhanced the activity of the ATP citrate lyase (ACL), which cleaved citrate into acetyl-CoA and oxaloacetate in both CBS 277.49 and WJ11 strains [29]. However, CBS 277.49 produced 2.1-fold more β-carotene at 48 h as compared to WJ11 in dark condition, indicating there is a significant difference in the distribution of acetyl-CoA for both fungal strains. It is noteworthy that the WJ11 is reported to be a superior lipid-producing strain (36%) in comparison to CBS 277.49, which produced only 15% lipid [29]. Our lipid analysis results also found that lipid production was greater in WJ11 (35% ± 0.49) as compared to CBS 277.49, which produced a maximum of 14.9% ± 0.28 lipids in our study (Figure 4d). Thus, lower β-carotene level in WJ11 might be due to the fact that acetyl-CoA pool in WJ11 was directed toward the lipid accumulation pathway rather than being used for β-carotene production, which resulted in lower β-carotene accumulation in this strain. On the other hand, most of the acetyl-CoA pool in CBS 277.49 strain might be channeled toward the mevalonate pathway, as this fungal strain could only produce a maximum production of 15% lipid, even at the most optimal condition [29].

As shown in Figure 4a, under the light condition, β-carotene production was enhanced up to 241.6 ± 0.53 μg/g and 112.69 ± 0.97 μg/g just after 6 h of exposure to white light for CBS 277.49 and WJ11, respectively. This result showed that light stress has a strong impact on β-carotene production, even before the nitrogen is exhausted in the media. After that, the pigment was gradually accumulated with the biomass increment at 48 h, and the highest pigment concentration 698.4 ± 3.68 μg/g was achieved at 48 h in CBS 277.49, which was 2.5-fold higher than in WJ11 (275 ± 1.34 μg/g). Similar to our findings, Zhang et al., obtained 667.3 μg/g of β-carotene accumulation in wild-type strain of CBS277.49 (MU241) upon continuous illumination, while investigating the regulatory pathway of β-carotene production. They also found a manifold increase in β-carotene production upon continuous illumination, as compared to the culture grown in dark similar to our study [7]. In another study, wild-type of *M. circinelloides* 277.49 produced 859 μg/g of β-carotene, using minimal agar and liquid media under light condition [32]. Khanafari et al. also found a threefold increase in β-carotene production when *M. hiemalis* was exposed to continuous white light for 72 h [33]. In our study we also found 2.7-fold increase in β-carotene accumulation for CBS 277.49 upon continuous illumination as compared to Dark. Similarly in WJ11, 2.2-fold increase in pigment accumulation was observed. This was consistent with the findings of previous studies that continuous illumination triggered pigment accumulation enormously as compared to fungal culture grown in dark [24,25]. Initial studies about photo-induction of carotenogenesis revealed the same trend in *Fusarium aquaeductuum*, i.e., exposure to light-induced a gradual carotenoid accumulation, reaching its maximum level at about 12 h after which synthesis kept increasing gradually for at least three days when the culture was maintained under continuous illumination [34]. Similar trend was not observed in our study i.e., we found maximum production at 48 h, after which decline in β-carotene accumulation was observed. However lipid production was not observed to be significantly affected by light and dark condition in our study as shown in (Figure 4c,d). This was in accordance to Zhang et al., who investigated the effect of 3 different light intensities and found that *Rhodotorula glutinis* culture, grown under either irradiation or without light did not show any significant difference in the total lipid accumulation [14].

In the present study after 48 h of cultivation, pigment production started to decline under light and dark conditions. This decline in β-carotene could be attributed to maximum lipid accumulation phase that is 72 h of cultivation as reported in our study and reported by [35] in *M. circinelloides*. It is well known fact that in *M. circinelloides*, FAS and mevalonate pathway shares the same acetyl-CoA pool, so it is speculated that after 48 h, more acetyl-CoA was diverted toward lipid metabolism as compared to carotenoid biosynthesis in our study. This could be one of the contributing factors for the decline in β-carotene accumulation after 48 h in both strains. It could be summarized as that after 48 h metabolic flux of carbon was pushed toward FAS pathway and pulled away from mevalonate pathway in our study.

Another aspect of higher β-carotene production in strain CBS 277.49 could be higher tricarboxylic acid cycle (TCA) activity. Higher TCA activity in strain CBS 277.49 was observed by Tang et al., in the comparative biochemical analysis of lipid production by CBS 277.49 and WJ11. They reported that activity of two important metabolic enzyme phosphofructokinase PFK and citrate synthase CS in strain CBS 277.49 was significantly higher than that of strain WJ11 in the lipid accumulation phase, suggesting greater carbon flux to TCA cycle in CBS 277.49, resulting in low lipid production in this strain (Figure 4d). Similarly in WJ11 this cycle was reported to be retarded, resulted in higher lipid production (36%) by fluxing more carbon to lipid synthesis pathway in this strain [29]. It has also been suggested previously that higher TCA cycle activity is responsible for generation of more ROS [36], which increased the accumulation of the β-carotene [36,37]. In one previous finding media was supplemented with a different intermediate of TCA cycle to investigate the regulation of carotenoid biosynthesis. They observed that these intermediates slowed down the activity of the TCA cycle, resulting in decreased production of ROS, which in turn reduced the production of carotenoid of interest [38]. Hence our results were consistent with these finding that increased accumulation of β-carotene could be attributed to more generation of ROS in CBS 277.49, which in turn is due to more active TCA cycle in CBS 277.49 as reported by Tang et al. [29]. Nevertheless, more study is required to support these claims. These facts supported our results to a greater extent for metabolic differences of these two strains in terms of β-carotene and lipid production.

### 2.3. Yield of Biomass, β-Carotene, and LIPID by CBS 277.49 and WJ11, under Light and Dark Conditions

The summary of the yield of Biomass, β-carotene, and Lipid production of two strains of *M. circinelloides* under light and dark at selected interval is presented in Table 1.

$$Y\;\mathrm{Biomass}=\frac{\mathrm{Biomass}}{Glucose\;consumed\;}\;g/L$$

$$Y\;\beta -\mathrm{carotene}=\frac{\beta -\mathrm{carotene}\;\left(\mu g/g\right)}{Glucose\;consumed\;g/L\;}$$

$$Y\;\mathrm{lipid}=\frac{\mathrm{Lipid}\;g/L}{Glucose\;consumed\;g/L\;}$$

### 2.4. Expression Levels of Key Genes Involved in β-Carotene Accumulation

In order to gain more insight into molecular nature of carotenogenic pathway at genetic level in both strains, RT-qPCR was carried out to analyze the mRNA level of two early genes of the general isoprenoid pathway (*hmgS* and *carG*) and two key genes of mevalonate pathway (*carB* and *carRP*) in *M. circinelloides* grown at 3, 12, 24, 48, and 72 h. Sample of 3 h was considered as the reference for calculation.

**Figure 5:**
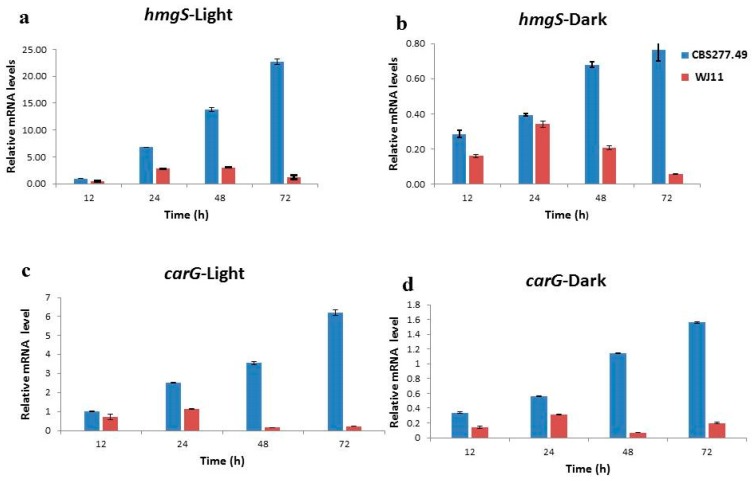

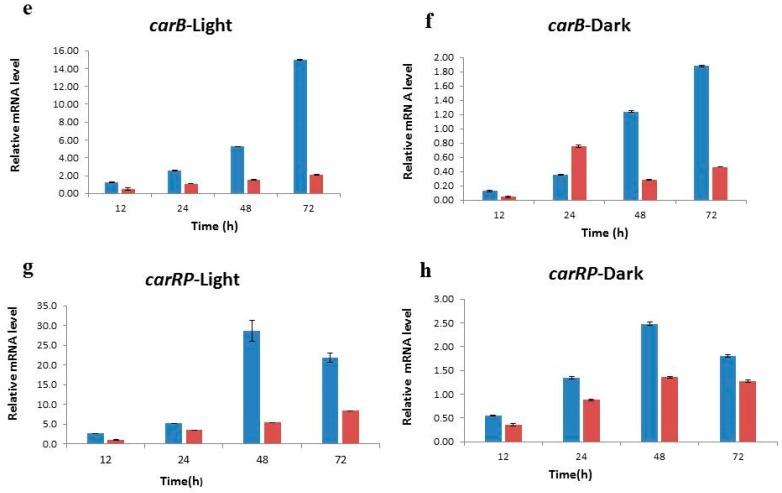
Determination of expression levels of four key genes involved in β-carotene synthesis pathway in CBS277.49 and WJ1. (**a**) *hmgS*-light), (**b**) *hmgS*-dark, (**c**) *carG*-light, (**d**) *carG*-dark, (**e**) *carB*-light, (**f**) *carG*-dark, (**g**) *carRP*-light, and (**h**) *carRP*-dark.

HMG-CoA synthase, considered as one of the key enzymes of the terpenoid biosynthesis catalyzes the conversion of acetoacetyl-CoA into 3-hydroxy-3-methylglutaryl-CoA (HMG-CoA) which acts as the first precursor for mevalonate synthesis. The sufficient supply of precursors in the mevalonate pathway is crucial for the accumulation of carotenoid [16]. The transcriptional level of *hmgS* was increased from 6 h, reaching its maximum value at 72 h for CBS 277.49, which was almost 18-fold higher in this strain as compared to WJ11. However, pattern of expression level in WJ11 was observed to be decreased after 48 h under light condition. The transcripts of *hmgS* under dark conditions had no significant expression though expression of *hmgS* was 2.5-fold higher in CBS 277.49 as compared to WJ11 at 48 h. In summarized form, the expression level of *hmgS* was increased from 48 to 72 h in CBS 277.49, but on contrary decreasing trend was observed for WJ11 during the same interval under both light and dark condition (Figure 5a,b). In conclusion, higher *hmgS* mRNA level in strain CBS 277.49 indicated more availability of HMG-CoA, thus more precursors were obtained by this strain than WJ11 for higher production of β-carotene.

Transcription of *carG* gene is increased in response to light [17]. In our study the expression level of *carG* was also found to be elevated throughout the cultivation period under light condition, i.e., its expression was upregulated in response to light stimulus from 12 to 72 h. Csernetics et al., also found an elevated level of *carG* in their overexpression study for the early genes of general isoprenoid under continuous illumination [16]. In our work *carG* transcripts increased gradually till 72 h in CBS 277.49 while in WJ11 the highest expression of *carG* was achieved at 24 h after which it started to decline. The transcript level for this gene was higher in CBS 277.49 than WJ11 under light condition. In dark condition, mRNA level of *carG* gene showed maximum expression at 72 h which was almost 7.8-fold higher in the former strain than the later one, although in WJ11 this gene showed decreasing trend with increasing cultivation time from 48 to 72 h. High expression of *carG* transcripts in CBS 277.49 indicated the superiority of this strain to produce more β-carotene over WJ11 due to more availability of GGPP as precursor for carotenoid synthesis (Figure 5c,d). It was consistent with findings of Matthäus et al., who reported the association of increased accumulation of carotenoid precursors with high expression of geranylgeranyl diphosphate synthase (ggs1) in *Yarrowia lipolytica* [13].

Transcription of structural genes for carotenoid biosynthesis increased enormously in response to light [21,22]. In our study expression level of *carB*, one of the structural genes of the carotenoid biosynthesis pathway also showed higher expression in *M. circinelloides*. The increasing trend in *carB* transcript was detected from 12 h in both strains under light condition reaching its maximum level at 72 h, which was approximately 7.5-fold higher in CBS 277.49 than WJ11 in continuous light situation. Meanwhile in dark condition, the expression level of *carB*, was low in both strains but comparatively higher in CBS 277.49 than WJ11. Again higher expression of this gene in CBS 277.49 strain proved higher capability to produce more β-carotene (Figure 5e,f).

High expression level of another structural gene *carRP* was detected throughout cultivation time. In light condition, its mRNA accumulation was highest among all 4 selected genes reaching its maximum value at 48 h in CBS 277.49, while in WJ11 maximum accumulation of *carRP* transcript was exhibited at 72 h, which was fivefold lower as compared to CBS 277.49. The association of light-induced carotenogenesis with the increased transcriptional level of two structural genes (*carB* and *carRP*) of carotene biosynthesis pathway has been demonstrated previously in *M. circinelloides* [21,22]. Under the dark condition, the transcription level of *carRP* in CBS 277.49 was almost threefold higher than in WJ11, of which it was almost below basal level (Figure 5g,h). Upregulation of these two structural genes in response to light stress and their higher expression even in dark may contribute to higher β-carotene production in CBS 277.49 than WJ11.

Our findings concluded that among all four key genes, the transcriptional level of *carG* was lower as compared to *carRP*, *hmgS*, and *carB* transcript levels throughout the cultivation period in both strains. Csernetics et al., also found a lower transcriptional level of *carG* as compared to other genes of the isoprenoid pathway in *M. circinelloides* [16]. The expression levels of investigated genes in present work were in such order *carRP* > *hmgS* > *carB* > *carG*. We found that overall the transcription level of *carRP* remained high, about twice of those of the other two genes and fourfold compared to *carG* expression level. Thus we speculated that higher expression of these four genes in CBS 277.49 as compared to WJ11 may account for the increased β-carotene production in this strain.

## 3. Materials and Methods

### 3.1. Microorganisms, Media, and Growth Condition

Wild type strains of *M. circinelloides* CBS 277.49 and WJ11 were used in this study. *M. circinelloides* (WJ11) was isolated from the soil at Jiangnan [29] while *M. circinelloides* f. lusitanicus (CBS 277.49) was provided by Prof. Victoriano Garre, Department of Genetics and Microbiology, University of Murcia, Spain [39]. Seed culture was prepared by inoculating 100 μL of spore suspension (approximately 10^7^ spores/mL) into 150 mL of K&R media (Kendrick and Ratledge medium) containing 30 g of glucose, 3.3 g of diammonium tartrate, 7 g of KH_2_P0_4_, 2 g of Na_2_HP0_4_, 1.5 g of MgS0_4_·7H_2_0, 1.5 yeast extracts, 0.1 g of CaCl_2_·2H_2_O, 8 mg of FeC1_3_·6H_2_0, 1 mg of ZnS0_4_·7H_2_0, 0.1 mg of CuSO_4_·5H_2_0, 0.1 mg of MnS0_4_·5H_2_0, and 0.1 mg of Co(NO_3_)_2_·6H_2_0 per liter, held in 500 mL baffled flasks and incubated at 28 °C for 24 h in shaking incubator at 150 rpm as described previously [40]. Then this seed culture was used to inoculate 1.5 L modified K&R media that contains 80 and 2 g/L diammonium tartrate held in 2 L fermenter for subsequent fermentation under dark and light condition. The dark condition was maintained by covering the fermenter by aluminum foil and light condition was provided by exposure of fermenter to three LED lamps. Each lamp provides intensity of 800 μmol/m^2^/s. The illuminance was measured by an illuminometer (tes-1339, Taiwan Taishi) [14]. Fermenters were controlled at 28 °C with stirring at 700 rpm and aeration at 1 *v/v* min^−1^. The pH was maintained at 6.0 by auto-addition of 2 M NaOH. Sampling was done at different time intervals e.g., 3, 6, 9, 12, 24, 48, 72, and 96 h.

### 3.2. Cell Dry Weight Determination (CDW)

Mycelia of both strains were collected at intended intervals from the fermenters for analysis. Biomass was harvested on a dried and pre-weighed filter paper by filtration through a Buchner funnel under reduced pressure and washed three times with distilled water, frozen overnight at −80 °C and then freeze-dried. The weight of the biomass was determined gravimetrically [35].

### 3.3. Estimation of Glucose and Nitrogen Consumption in the Culture Supernatant

The rate of glucose consumption was determined from liquid supernatant by glucose oxidase kit and ammonium utilization by culture was determined by the indophenol method [41].

### 3.4. Extraction and Analysis of Total Fatty Acids in Cell

For extraction of total lipid biomass of 6, 12, 24, 48, 72, and 96 h was taken and extraction was done by Folch method as described previously [42], with some minor modification in this study, i.e., 50 mg of dried mycelia were used in our study.

### 3.5. Extraction and Analysis of β-Carotene

β-carotene was extracted from mycelia powder as described previously [5] with some minor modification in our study. A total of 100 mg of mycelia powder was dissolved in 700 μL of hexane, followed by vortexing. This extraction step was repeated until the mycelia powder became colorless. Then extracts were collected in 15 mL falcon tube, combined and partitioned in an equal volume of 10% diethyl ether in petroleum ether. Next, 2 mL of distilled water was added to facilitate the separation of two layers and centrifuged at 3200 rpm (8 min, 4 °C). The petroleum ether fraction was dried using nitrogen. 

High-performance liquid chromatography (HPLC) was performed for β-carotene standard (sigma) and samples. Dried extracts were resuspended in 700 μL tetrahydrofuran supplemented with butylated hydroxytoluene (100 μg/mL) and subjected to HPLC analysis directly. Next, 10 μL of samples were analyzed by HPLC on an infinity Lab Proshell 120 EC-C18 column (4.6 × 150, ODS 4 μm). Two solvents A (96% methanol) and B (100% methyl-terc-butyl ether) were used as mobile phase. These two solvents were used in following gradient to analyze our samples: min/solvent A_%_/solvent B_%_ was 0/99/1; 8/60/40; 13/46/54; 15/0/100; 18/0/100; 21/99/1; 25/99/1) at a flow rate of 1 mL/min. Column thermostat temperature was set as 35 °C and detection wavelength was set as 450 nm, using diode-array detector (Agilent Technologies, Santa clara, CA, USA).

### 3.6. Blast Analysis

From genomic data of *M. circinelloides* CBS 277.49 available at JGI, we got sequence of four key genes that are involved in β-carotene biosynthesis, e.g., *hmgS* (JGI accession number (ID 51849, 1812 bp), *carG* (ID 155025, 1293 bp), *carB* (ID 31317, 1864 bp), and *carRP* (ID 154743, 2093 bp). Based on homology sequences and blast analysis we also identified these four genes in WJ11 genome (available at NCBI). Percent identity and similarity were determined by BLAST nucleotide sequence alignments (Table 2).

### 3.7. RNA Extraction and RT-qPCR

Strains were grown in a 2 L fermenter with modified K&R medium, and the mycelium was harvested at 3, 6, 12, 24, 48, and 72 h. Extraction of total RNA of *M. circinelloides* was done by using TRIzol after disruption of biomass in pestle and mortar, using liquid nitrogen. In order to investigate mRNA levels of four key genes of the carotenogenic pathway, RT-qPCR was conducted. Reverse transcription of RNA was performed by using the Prime ScriptRT reagent kit (Takara Biotechnology, Dalian Co., Ltd, Dalian, China) according to the manufacturer’s instructions. Primers were designed according to RT-qPCR requirement (Supplementary Table S1), and RT-qPCR was performed by using these primers on Light Cycler 96 Instrument (Roche Diagnostics GmbH, Switzerland) with FastStart Universal SYBR Green Master (ROX) Supermix (Roche) as instructed by the manufacturer. The mRNA expression level was normalized to the level of actin gene, and the results were expressed as relative expression levels. The data were quantified by the method of 2^−ΔΔCt^ [7].

## 4. Conclusions

*M. circinelloides* strain CBS 277.49 accumulated up to 698.4 ± 3.68 μg/g of β-carotene, whereas strain WJ11 accumulated 275.06 μg/g, when cultivated under continuous illumination, using K&R media. HPLC chromatogram analysis showed higher β-carotene production in light condition as compared to dark condition for both strains. In high-β-carotene-producing strain CBS 277.49, all selected genes were found to have high mRNA levels as compared to low-pigment-producing strain WJ11. It was suggested by RT-qPCR analysis that, in CBS 277.49, light stress helped in elevation of transcription of all selected genes. Hence, our results concluded that metabolic network in CBS 277.49 is channeling more acetyl-CoA toward the mevalonate pathway rather than the FAS pathway, indicating more availability of precursors for β-carotene production. In conclusion, CBS 277.49 can be used for the production of β-carotene on an industrial scale by advanced biotechnological processes (genetic manipulation and media optimization), owing to the availability of efficient genetic tools and a simpler fermentation process as compared to other zygomycetes.

## Figures and Tables

**Table 1:** Determination of yield of biomass, β-carotene and lipid production by CBS 277.49 and WJ11 under Light condition and Dark condition.

**H**	**Light-CBS 277.49**			**Light-WJ11**		
	**Y Biomass**	**Y β-Carotene**	**Y Lipid**	**Y Biomass**	**Y β-Carotene**	**Y Lipid**
6	0.10	17.90	0.003	0.26	9.80	0.01
12	0.11	11.71	0.005	0.25	10.12	0.03
24	0.16	17.76	0.01	0.28	7.53	0.06
48	0.16	12.07	0.02	0.22	5.33	0.07
72	0.14	5.59	0.02	0.21	4.04	0.07
96	0.14	4.04	0.02	0.19	3.22	0.06
**H**	**Dark-CBS 277.49**			**Dark-WJ11**		
	**Y Biomass**	**Y β-Carotene**	**Y Lipid**	**Y Biomass**	**Y β-Carotene**	**Y Lipid**
6	0.13	2.0	0.003	0.24	0.91	0.01
12	0.13	3.3	0.005	0.29	1.45	0.03
24	0.22	7.2	0.02	0.29	3.25	0.06
48	0.18	4.2	0.02	0.25	2.33	0.08
72	0.16	2.7	0.02	0.24	1.92	0.08
96	0.16	2.4	0.02	0.21	1.41	0.07

**Table 2:** Identification of key genes analyzed in this study by RT-qPCR.

Gene	CBS 277.49 (Id)	WJ11 (Scaffold)	% Identity	% Similarity
HMG-CoA synthase-*hmgS*	51849	scaffold00204.8	86.06	79
GGPP synthase-*carG*	155025	scaffold00277.10	81.82	85
Phytoene dehydrogenase-*carB*	31317	scaffold00226.18	85.86	82
Phytoene-synthase/Lycopene cyclase-*carRP*	154743	scaffold00226.19	81.34	95

## Supplementary Materials

The following are available online at https://www.mdpi.com/2218-1989/10/1/38/s1. Supplementary Table S1. Primers sequences used in this study.
