# Supplementary material for: Comparative Analysis of β-Carotene Production by Mucor circinelloides Strains CBS 277.49 and WJ11 under Light and Dark Conditions
Source: Metabolites. 2020 Jan 16;10(1):38. doi: 10.3390/metabo10010038 (PMC7022308; doi:10.3390/metabo10010038)
Supplement: Supplementary file 1 [file metabolites-10-00038-s001.pdf]

Table S1. Primers used in the RT-qPCR analysis.

Primer	CBS 277.49 (5′-3′)	WJ11 (5′-3′)
carG-F	CTAAAGGATTTCGTCATTTGCCCG	GTGGAGTTCAGTAGGACATGTCAAG
carG-R	GATTTGGACATGACATTTTCTCCCG	GATCGACGATGTGCAGGACGATTC
hmgS-F	CATCCAAAGATATTGTGTTGCGAG	CGTTGTAAGATGCACGAGCAAAAGAC
hmgS-R	GACGGAAACCACGGGATACCATTAG	CTATCGTCGTTGAGCGTGGTCTC
carB-F	TATGGGCATGTCGCCCTACG	GGTGTGTGCTTGCACCAACAAAG
carB-R	AGATCTGCGTTACACACAACCGC	ACCACCGAGAATTATCCAGCCATG
car*RP*-F	CAAGGCTTGGCATTTGGCG	AAAGCTTGGCATTTAGCGGTCCC
car*RP*-R	CATCGACCCAGCAGAGAAACAG	GAATTCTTCCACGGGCAGGTGAG
Actin-F	GATGAAGCCCAATCCAAGAGAGGT	GATGAAGCCCAATCCAAGAGAGGT
Actin-R	TCTTCTCACGGTTGGACTTGGG	TCTTCTCACGGTTGGACTTGGG
